# Books and Pamphlets Received

**Published:** 1887-09

**Authors:** 


### BOOKS AND PAMPHLETS RECEIVED.

*Beautiful Songs.* A new and choice collection of Songs for the Sunday Schools ; also a responsive service for each month in the year. By S. W. Straub, Chicago. 1887.

In this day of Sabbath schools, where so many good men of every name and profession are engaged in the Sabbath school work, we take pleasure in recommending this excellent selection of songs. It contains about 300 choice pieces, both new and old. Price, 35 cents single copy, or $3.60 per dozen.

*Elements of Botany*, incluidng Organography, Vegetable Histology, Vegetable Physiology and Vegetable Laxonomy, and a glossary of botanical terms, illustrated by nearly five hundred engravings from drawings by the author. By Edson S. Bastin, A.M., F.R.M.S., Professor of Botany, Materia Medica and Microscopy in the Chicago College of Pharmacy. Chicago, G. P. Engelhard & Company. 1887.

We have here a work on Botany of 282 oc. pages, ably written by an experienced teacher, extensively illustrated and tasteful in its typographical execution.

With all the books extant on this interesting subject, there is yet need of a work less technical, adapted to the ordinary reader as well as to the student, and suited alike to the high schools and to the colleges of pharmacy and of medicine. This work claims to accomplish these special objects, and will therefore meet a want which has been felt for some time past.

While the technical names are given, the common name is also made known as far as possible, the descriptive terms have been explained, and a glossary has been appended which greatly aids the student. This work may be classed as among the best and most recent works now extant on the subject of Botany.

*A Manual of Midwifery.* By Alfred Lewis Galabin, M.A., M.D., Obstetric Physician and Lecturer on Midwifery and the Diseases of Women to Guys' Hospital; Examiner in Midwifery to the Conjoint Examining Board for England; Fellow of the Royal College of Physicians, London; late Fellow of Trinity College, Cambridge. Illustrated with 227 wood engravings. Philadelphia: P. Blakiston, Son & Co., 1012 Walnut street. 1886.

We have here a book which, though a manual in point of size, yet includes all that is now known of importance on the subject treated. In the main, the work agrees with the most approved authorities, but contains useful original suggestions. The chapter on puerperal fevers will be found unusually interesting. The favorable results from the high forceps operation in contracted pelvis are worthy of special attention. Upon the whole the book is well up on important points, and is a very useful and practical work.

*What to do in Cases of Poisoning.* By William Murrell, M.D., F.R.C.P.; Lecturer on Pharmacology and Therapeutics in the Westminister Hospital; Examiner in Materia Medica in the University of Endinburg, and to the Royal College of Physicians of London, etc. First American from the fifth English edition. Edited by Frank Woodbury, M. D., Fellow of College of Physicians of Philadelphia; Professor of Materia Medica, Therapeutics and Clinical Medicine in Medico-Chirurgical College of Philadelphia. Published by the Medical Register Co. Philadelphia.

A work of 158 pp. The author remarks of the work that he has been "told it has been the means of saving many lives * *. A complaint has been made that the book is getting too big. I admit it; but the fact is, there are too many poisons now-a-days." We regard it as a very useful and practical book.

*A Pocket Dictionary of the English Language;* compiled from the quarto and school dictionaries of Joseph E. Worcester, LL. D. By Loomis J. Campbell, with foreign words and phrases, abreviations, rules for spelling. Profusely illustrated. Philadelphia: J. B. Lippincott Company.

An excellent and most useful little work.

*Syphilis.* By Johnathan Hutchison, F.R.S., LL.D., with eight chromo-lithographs. Philadelphia: Lea Brothers & Co., Publishers.

We regard this as an able and practical work, very useful to the practitioner who not having time to consult the many works on this subject can here find the pith and cream of that which is best. The work, though condensed in its statements, is sufficiently full and comprehensive, covering all essential and important points connected with this interesting subject.

*Public Health* The Lombe Prize Essays. Award made at the Thirteenth Annual Meeting of the American Public Health Association, Washington, D. C., Dec. 19, 1885, with an Appendix. Second edition. Concord, N. H. Republican Press Association. 1886.

A book of 198 large octavo pp., containing articles as follows:

1. On Healthy Homes and Food for the Working Classes, by Victor C. Vaughn, Ph D, M. D.; Professor in University of Michigan.

2. The Sanitary Conditions and Necessities of School Houses and School Life, by D. F. Lincoln, M. D., Boston, Mass.

3. Disinfection and Individual Prophylactics against Infectious Diseases, by George M. Sternberg, M D., Major and Surgeon U. S. A.

4. Preventable Causes of Disease, Injury and Death in American Manufactories and Workshops, and the Best Means for Preventing and Avoiding Them, by George H. Joelond, Springfield, Mass.

*Sexual Impotence in the Male and Female* By William A. Hammond, M. D., Surgeon-General U. S. Army (retired list); Professor of Diseases of the Mind and Nervous System at the New York Post-Graduate Medical School, etc. Detroit: G. S. Davis. 1887.

This work is presented to us in this the second edition in a neat, bold and excellent type. Containing, in addition to impotence in the male as in the first edition, a consideration of the same disorder in the female. There are 385 pp. of ably written and very intsructive matter on a subject of great importance and but little understood. The subject is treated under the following general, heads, the subdivisions in each covering a wide field.

1. Absence of sexual desire.
2. Absence of the power of erection and consequent intromission.
3. Absence of the power of ejaculating
4. Absence of the ability to experience pleasure during the act of copulation.

The second section of the work referring to the female is treated under the following heads, to-wit:

1. Absence of sexual desire due to absence of arrest of development of the clitoris. Orgin of sexual desire.
2. Inability by reason of pathological obsticles to entrance, etc.
3. Vaginism.
4. Absence of the ability to experience the sexual orgasm.

*Science.* The *Philadelphia Press*, in referring to this journal, says: "We know of no weekly scientific journal in English, at home or abroad, which so fully meets the general needs of that large class in civilized countries who touch science at many points without mastering it at any." Every one interested in political science, educational science, or mental science should subscribe. $3.50 per year; trial subscription, four month $1.00. N. D. C. Hodges, 47 Lafayette Place, New York.

